# Can tobacco dependence provide insights into other drug addictions?

**DOI:** 10.1186/s12888-016-1074-4

**Published:** 2016-10-27

**Authors:** Joseph R. DiFranza

**Affiliations:** Department of Family Medicine and Community Health, University of Massachusetts Medical School, 55 Lake Avenue North, Worcester, MA 01655 USA

**Keywords:** Addiction, Adolescents, Nicotine, Smoking, Tobacco, Dependence

## Abstract

Within the field of addiction research, individuals tend to operate within silos of knowledge focused on specific drug classes. The discovery that tobacco dependence develops in a progression of stages and that the latency to the onset of withdrawal symptoms after the last use of tobacco changes over time have provided insights into how tobacco dependence develops that might be applied to the study of other drugs.

As physical dependence on tobacco develops, it progresses through previously unrecognized clinical stages of wanting, craving and needing. The latency to withdrawal is a measure of the asymptomatic phase of withdrawal, extending from the last use of tobacco to the emergence of withdrawal symptoms. Symptomatic withdrawal is characterized by a wanting phase, a craving phase, and a needing phase. The intensity of the desire to smoke that is triggered by withdrawal correlates with brain activity in addiction circuits. With repeated tobacco use, the latency to withdrawal shrinks from as long as several weeks to as short as several minutes. The shortening of the asymptomatic phase of withdrawal drives an escalation of smoking, first in terms of the number of smoking days/month until daily smoking commences, then in terms of cigarettes smoked/day.

The discoveries of the stages of physical dependence and the latency to withdrawal raises the question, does physical dependence develop in stages with other drugs? Is the latency to withdrawal for other substances measured in weeks at the onset of dependence? Does it shorten over time? The research methods that uncovered how tobacco dependence emerges might be fruitfully applied to the investigation of other addictions.

## Background

Longitudinal studies of tobacco dependence in adolescents have revealed surprising features of this condition that had remained hidden from researchers for more than 50 years. Even within the field of addiction research, investigators tend to operate within their own silos of interest. This review will focus primarily on an explanation of recent advances in our understanding of the natural history of the developmental stages of tobacco dependence. Some of the similarities and differences between tobacco dependence and other addictions will be noted. The essay will conclude with suggestions on how researchers might apply these new insights to the study of other addictions.

## Main text

### The natural history of tobacco dependence

Nicotine is among the least rewarding of all addictive drugs: only 20 % of first time users find smoking to be relaxing [1]. Smoking provides an image, a shared social activity, and something to do when bored. These factors motivate smoking until dependence takes over. Prior to the onset of dependence, sporadic non-daily use is typical.

#### The classic withdrawal syndrome

The seminal research on tobacco withdrawal focused on individuals with advanced dependence [2–4], as was the case with research on withdrawal associated with other substances [5, 6]. Because of this history, the term ‘withdrawal’ has traditionally been used to describe the symptoms experienced by individuals with advanced physical dependence [7, 8]. In this report, the historical connotation of the word ‘withdrawal’ is expanded to accommodate recent advances in scientific knowledge afforded by the study of individuals with emerging dependence.

#### The emergence of physical dependence

Many addictive drugs cause a physical (physiologic) dependence that manifests as withdrawal symptoms. [7] (The term physical dependence is introduced here to indicate that the discussion is about to focus on that aspect of dependence related to withdrawal phenomena. The author acknowledges that there are psychological aspects to dependence and that the physical and psychological may be tightly entwined.) Tobacco withdrawal symptoms are identifiable as such because (1) they recur upon each withdrawal from tobacco, (2) they appear in a characteristic sequence, (3) they appear after a characteristic latency, and (4) they are relieved immediately upon using tobacco. Based on these criteria, it has been shown that physical dependence on tobacco develops through a characteristic sequence of stages in all addicted smokers [9].

As physical dependence begins to develop, the earliest symptom is withdrawal-induced ‘wanting’ of a cigarette [10]. ‘Wanting’, by definition, is mild, short-lived and fairly easy to ignore. It does not intrude upon the patient’s thoughts. For individuals in the first stage of physical dependence, wanting is the only withdrawal symptom experienced. The second stage of physical dependence is characterized by withdrawal-induced ‘craving’. Craving is a more intense and persistent sensation than is wanting, but what distinguishes craving qualitatively from wanting is the fact that craving intrudes upon the patient’s thoughts. Smokers often describe craving as if something inside of their head is telling them that it is time to smoke. The third and final stage in the development of physical dependence is withdrawal-induced ‘needing’. The needing stage is characterized by a desire to smoke that is so intense and urgent that it cannot be ignored and the individual is so distracted that he or she is unable to function normally [10]. In the words of one teen, “You really want one. You know you need it. You know you’ll feel normal after smoking, and you have to smoke to feel normal again” [10]. When smokers report that they need to smoke, it is not a need to experience pleasure, it is an acute and urgent need to relieve withdrawal symptoms [10]. This description of the Wanting-Craving-Needing stages is not based on, or predicted by any specific theory of addiction, it is a clinical description based on case histories, validated by larger surveys [9–12]. To the author’s knowledge, stages in the development of physical dependence on other drugs have not been identified.

Traditionally, it had been assumed that long-term heavy daily use of tobacco was a prerequisite to dependence [13], and the DSM has suggested that prolonged heavy use was a prerequisite for withdrawal syndromes generally [14]. However, in a national survey, 16 % of adolescents who used tobacco only one or two days per month reported strong cravings to use tobacco, and this symptom of physical dependence increased proportionate to the frequency of tobacco use to 78 % among those who had initiated daily use [15]. Irritability and restlessness during withdrawal were reported by 13 % of adolescents that used tobacco 1–2 days/month and by 70 % of daily users [15].

Close to two dozen peer-reviewed studies document symptoms of physical tobacco dependence in nondaily or very light daily smokers [16]. Symptoms can appear after only a few uses of tobacco. [17] Studies demonstrate that physical withdrawal symptoms can be elicited by the administration of an opioid receptor antagonist after a brief exposure to morphine [18, 19]. These studies raise the possibility that clinically significant physical dependence on other drugs is present in some form soon after the onset of infrequent use. If so, we should not expect their symptoms to be identical to those seen during withdrawal among individuals with far advanced dependence. It is likely that only a mild ‘wanting’ to use the drug manifesting at a predictable interval after the last use might be the first sign of physical dependence.

Wanting has not been previously recognized as a withdrawal symptom, probably because of the perceived difficulty in distinguishing withdrawal-induced wanting from the wanting that we all experience as a part of daily living. The key to recognizing withdrawal-induced wanting is to apply the following criteria. Does the wanting recur predictably upon each withdrawal from the drug? Does it appear after a characteristic latency (time interval)? Is it relieved immediately upon using the drug? Is it followed by other symptoms in a predictable sequence?

As physical dependence develops in new smokers, the symptoms of wanting, craving and needing develop in that order. During each episode of withdrawal, symptoms emerge in this same order: wanting, then craving, then needing. For individuals in the first stage of physical dependence, the desire to smoke never proceeds beyond wanting. For those in the second stage, the desire to smoke never proceeds beyond craving, while for those in the third stage, the desire to smoke will always progress to needing if abstinence is maintained. Thus, as physical dependence develops, it progresses through stages of wanting, craving and needing, and during each episode of withdrawal, symptoms escalate from a wanting phase, to a craving phase, to a needing phase. Table 1 presents a validated measure of the stages of physical dependence [20]. Individuals answer the three questions and the stage or level of physical dependence is determined by the most advanced symptom endorsed.

**Table 1:** A clinical measure of the Stages of Physical Dependence

	Describes me not at all	Describes me a little	Describes me pretty well	Describes me very well
If I go too long without smoking, the first thing I will notice is a mild desire to smoke that I can ignore.				
If I go too long without smoking, the desire for a cigarette becomes so strong that it is hard to ignore and it interrupts my thinking.				
If I go too long without smoking, I just can’t function right, and I know I will have to smoke just to feel normal again.				

Individuals answer the three questions and the stage or level of physical dependence is determined by the most advanced symptom endorsed. The first item measures wanting, the second craving, and the third needing

The classically recognized symptoms of nicotine withdrawal such as irritability, anxiety, restlessness, moodiness, impatience, difficulty concentrating and trouble sleeping [7, 8], do not appear in a standard order as physical dependence develops but are usually associated with the needing stage of physical dependence and the needing phase of withdrawal. In practical terms, this indicates that the seminal work on tobacco withdrawal focused on individuals who had already advanced to the needing stage. Earlier stages of physical dependence were not discovered for many decades because light smokers had been systematically excluded as research subjects. Seminal studies on other withdrawal syndromes have similarly focused on treatment seeking individuals with advanced dependence [2, 3]. This raises the question as to whether similar clinical stages of wanting-craving-needing in the development of physical dependence to other drugs have gone unnoticed because of the natural tendency to focus on treatment seeking individuals with the most advanced dependence.

Table 2 lists symptoms selected by the DSM as criteria for a tobacco withdrawal syndrome [7]. As this list is limited to symptoms that would be experienced only by individuals at the needing stage, the DSM criteria are not a sensitive indicator of physical dependence.

**Table 2:** DSM-5 tobacco withdrawal criteria [7]

DSM-5 tobacco withdrawal criteria	Comments
A. Daily use of tobacco for at least several weeks.	But the DSM text notes that withdrawal occurs in nondaily smokers.
B. Abrupt cessation of tobacco use, or reduction in the amount of tobacco use, followed within 24 h by four (or more) of the following signs and symptoms: Irritability, frustration, or anger. Anxiety. Difficulty concentrating. Increased appetite. Restlessness. Depressed mood. Insomnia.	Smokers with a latency to withdrawal of greater than 24 h do not experience withdrawal symptoms within 24 h.
C. The signs or symptoms in Criteria B cause clinically significant distress or impairment in social, occupation, or other important areas of functioning.	Tobacco withdrawal symptoms are rarely severe enough to preclude normal occupational functioning.
D. The signs or symptoms are not attributed to another medical condition and are not better explained by another mental disorder, including intoxication or withdrawal from another substance.	Experienced smokers would never be confused as to the cause of the listed withdrawal symptoms as they would simultaneously experience craving for tobacco.

The stage of physical dependence, as measured by the instrument in Table 1, correlates with progressive changes in the neural architecture of the anterior cingulate gyrus [21, 22]. As the stage of physical dependence advances, neural pathways between the anterior cingulate gyrus and the precuneus increase in number, while those linking to the frontal lobe decrease substantially. Neural activity in networks involving the anterior cingulate increases in proportion to the strength of craving reported by individuals in withdrawal [22, 23]. Although the symptoms of tobacco withdrawal are primarily psychological, the correlation of both the stage of physical dependence and the intensity of withdrawal-induced craving with measures of brain structure and function confirm that physical processes are involved in the development and expression of physical dependence on tobacco. [21, 22] Changes in neural architecture have also been identified in conjunction with other forms of addiction [24, 25]. The progressive changes in neural architecture in parallel with stages of symptom development suggest that some of these changes represent neural adaptation rather than non-specific toxicity [26]. The identification of these changes among very light smokers suggests that it would be worthwhile looking for neural changes among users of other drugs who do not show the classic signs of physical dependence. As with the clinical research, imaging research has missed the opportunity to identify the progression in changes because studies have focused on individuals with advanced dependence.

#### The latency to withdrawal

The latency to withdrawal is a measure of the time elapsed between the last use of tobacco and the onset of withdrawal symptoms. [10, 27, 28] As the symptoms of wanting, craving and needing emerge in that sequence during withdrawal, there is a latency to wanting, a latency to craving, and a latency to needing.

The latencies vary between individuals, ranging from 4 weeks to as short as several minutes [27–29]. An 18 year-old woman reported *wanting* a cigarette after 30 min but being able to go two days before she absolutely *needed* one, while a 19 year-old man would *want* a cigarette in 2–3 h, and *need* one in 6–7 h [10]. The observation that latencies in some smokers are measured in weeks, while in others they are measured in minutes, reflects the fact that the latencies shorten with repeated exposures to nicotine [10, 29].

Since the short half-life of nicotine results in its elimination from the body within a day, it may seem paradoxical that withdrawal symptoms may not appear in novice smokers until several weeks have passed since their last cigarette. The mechanism that triggers tobacco withdrawal symptoms is unknown, but the idea that it is triggered by nicotine levels dropping below a threshold level is incompatible with the clinical evidence. Similar to the phenomenon whereby delirium tremens may not be experienced until several days after alcohol has been cleared from the blood [3], newly addicted smokers may not experience withdrawal symptoms until weeks after their last cigarette [10, 27–29]. In contrast, chain smokers report the need to smoke within minutes of having smoked [27, 28] while blood nicotine levels are still quite high [30]. Nicotine alters the expression of 162 genes in the brains of adolescent rats [31], triggers the release of a half-dozen neurotransmitters [32–35], and alters the production of neurotransmitters and neuronal responsivity for up to four weeks following a single dose [34, 36]. It is likely that mechanisms other than nicotine levels trigger withdrawal. Since nicotine withdrawal in humans is not triggered by the administration of an antagonist as is the case with opiate withdrawal, the mechanisms responsible for withdrawal symptoms may be drug-specific. Admittedly, this suggests that lessons learned about tobacco addiction may not apply to other drugs. On the other hand, non-addictive drugs can produce withdrawal symptoms, so some withdrawal symptoms may have little relevance to addiction, suggesting that differences in withdrawal mechanisms between drugs may not be relevant.

#### Shortening latencies and the trajectory of tobacco use

The clinical implication of shortening latencies is that the length of time an individual remains comfortable after putting out a cigarette decreases over time. This increases the frequency at which cigarettes must be smoked to maintain comfort. [28] An increase in the frequency of smoking is one of the earliest signs of dependence [37]. At a latency to craving of two days, a person could keep withdrawal at bay by smoking one cigarette every other day. Fifteen cigarettes would be sufficient to keep withdrawal at bay for 30 days. But when the latency to craving shortened to 45 min, one would have to smoke every 45 min to keep withdrawal at bay, which would entail smoking >500 cigarettes over a 30 day period.

A 21 year-old woman described a latency-to-craving of two days after having smoked for about six weeks at age 16. Her latency decreased to four hours by age 16½, to two hours by age 17, to 1.5 h by age 18, to one hour by age 19, and to 30–45 min by age 21. Over this time, her intake increased from 5 cigarettes/day to 15 [10]. Individuals addicted to opiates do not have to use their drug with such frequencies, but is it possible that at the onset of opiate dependence, a single dose may keep withdrawal symptoms at bay for many weeks, but a shortening of the latency to withdrawal demands a gradual escalation in the frequency of use?

Aware of their latencies, smokers sometimes smoke in anticipation of a period of abstinence to postpone the onset of withdrawal (e.g., prior to going to sleep, school or work). Even addicted smokers can smoke for pleasure, or to relieve stress or boredom. This is reflected in the fact that cigarette consumption correlates only moderately with the latencies (rho = −.53, *r* = −.53, and Kendall’s tau b = −.53 in three studies of adolescents) [27–29]. This level of correlation indicates that only a proportion of cigarettes are smoked for the purpose of relieving withdrawal symptoms. Physical dependence need not be the only reason why addicted individuals self-administer their drug of choice, but it may put an outside limit on how long they can comfortably refrain from use.

A new withdrawal period begins each time a smoker finishes a cigarette. From the moment the cigarette is finished the timer starts on the latency period during which withdrawal symptoms are not experienced. This asymptomatic phase of withdrawal may last from a few minutes to several weeks. The asymptomatic phase of withdrawal is followed by a symptomatic withdrawal phase during which wanting, craving, needing and the DSM withdrawal symptoms (anger, anxiety, restlessness, etc.) emerge. The act of smoking aborts one episode of withdrawal and initiates another, analogous to hitting the snooze button on an alarm clock.

Traditionally, when researchers referred to withdrawal they were referring to advanced withdrawal *symptoms*, as described in the DSM. However, under this new conceptualization of withdrawal, smokers are in a state of withdrawal (asymptomatic or symptomatic) anytime they are not actively smoking. In other words, the term ‘withdrawal’ is used to describe a physiologic state rather than specific symptoms. Analogously, when the cause of Acquired Immune Deficiency Syndrome was unknown, symptomatic individuals were diagnosed with AIDS. After the underlying cause was better understood, individuals were described as carrying the human immunodeficiency virus, which is often an asymptomatic state. Smokers are in a state of withdrawal anytime they are not smoking. If they go too long without smoking they may experience withdrawal symptoms of increasing severity as time passes. Individuals with short latencies may experience symptomatic withdrawal dozens of times each day even though they are making no effort to maintain abstinence.

Shortening latencies compel smoking at progressively shorter intervals and the resulting escalation in cigarette consumption can be mathematically modeled as a smooth trajectory [38–40]. The latencies shorten at different speeds and to different degrees in different individuals, determining whether that person will plateau as a light, moderate or heavy smoker. Longer latencies allow some physically dependent smokers to remain light smokers over a lifetime [41]. In others, the latencies shorten so much that they feel compelled to chain smoke. The fact that latencies shorten is the key to understanding the pathophysiology of tobacco dependence, its behavioral manifestations and its clinical course throughout the lifespan [42]. To the author’s knowledge, latencies have not been systematically studied in relation to any other drugs.

#### Clinical manifestations of shortening latencies

The trajectory of the frequency of tobacco use is very different from that of a drug like alcohol. When adolescents are drinking to achieve intoxication, the amount of alcohol consumed on weekends may increase substantially without a concomitant increase in the number of drinking days per week. With tobacco, the opposite pattern is seen. As the latency shortens, smoking frequency increases from monthly to weekly, to several days per week, and finally to daily smoking. All the while, the number of cigarettes smoked on smoking days remains around one to two [43]. Only when the latency shortens from days to hours is there a gradual increase in the number of cigarettes smoked per day [44]. Individuals with very short latencies will begin to feel uncomfortable within minutes of finishing a cigarette, prompting chain smoking. Such individuals will spend a great deal of time smoking (Table 3, Diagnostic and Statistical Manual 5 (DSM 5) criterion 3).

**Table 3:** DSM 5 criteria [7]

DSM 5 criteria	Comments in relation to tobacco use
A problematic pattern of tobacco use leading to clinically significant impairment or distress	
1. Tobacco is taken in larger amounts or over a longer period than was intended.	As benders do not occur with tobacco, this criterion is met when the user has failed in an attempt to quit or cut down.
2. There is a persistent desire or unsuccessful efforts to cut down or control tobacco use.	The user has failed in an attempt to quit or cut down.
3. A great deal of time is spent in activities necessary to obtain or use tobacco.	Such as chain smoking, or minors loitering in front of a store asking adults to buy tobacco for them.
4. Craving, or a strong desire or urge to use tobacco.	This criterion would be met by individuals at the craving or needing stages of physical dependence.
5. Recurrent tobacco use resulting in a failure to fulfill major role obligations at work, school, or home.	As tobacco is not intoxicating, this criterion is not particularly relevant to tobacco.
6. Continued tobacco use despite having persistent or recurrent social or interpersonal problems caused or exacerbated by the effects of tobacco (e.g., arguments with others about tobacco use).	
7. Important social, occupational, or recreational activities are given up or reduced because of tobacco use.	This would typically happen when a short latency to withdrawal makes a person uncomfortable when smoking is not allowed.
8. Recurrent tobacco use in situations in which it is physically hazardous (e.g., smoking in bed).	
9. Tobacco use is continued despite knowledge of having a persistent or recurrent physical or psychological problem that is likely to have been caused or exacerbated by tobacco.	Continued use generally reflects failed attempts at cessation.
10. Tolerance, as defined by either of the following:	
a. A need for markedly increased amounts of tobacco to achieve the desired effect.	As tobacco is not intoxicating, this criterion does not apply to tobacco use.
b. A markedly diminished effect with continued use of the same amount of tobacco.	A shortening of the latency to withdrawal indicates that a cigarette has a markedly diminished effect on sustaining the asymptomatic phase of withdrawal.
11. Withdrawal, as manifested by either of the following:	
a. The characteristic withdrawal syndrome for tobacco. (See Table 2)	Physical dependence can be present long before it is of sufficient severity to cause at least 4 withdrawal symptoms.
b. Tobacco (or a closely related substance, such as nicotine) is taken to relieve or avoid withdrawal symptoms.	Wanting, craving and needing are withdrawal symptoms. Smoking in response to these symptoms indicates smoking to relieve withdrawal.

As the latency shortens, smokers may experience difficulty in refraining from smoking in situations where it is not allowed and may avoid such situations because the emergence of craving and needing makes them uncomfortable (Table 3, DSM 5 criterion 7). Individuals with latencies shorter than the time spent in bed may smoke just before going to bed and still feel the need to smoke immediately upon arising. The latency explains why the time to the first morning cigarette is a valid measure of dependence [15, 45]. Insomnia is a symptom of tobacco withdrawal and individuals with very short latencies may awaken during the middle of the night needing to smoke [46]. The discomfort of individuals in the needing stage of withdrawal may be such that they will smoke even when sick in bed because the discomfort of withdrawal makes them feel even worse, and why, even some nondaily smokers feel compelled to go outside in severe weather to smoke [37].

#### A note on medical and psychiatric approaches to diagnosis

The medical approach to identifying and diagnosing conditions is based on evidence of a disruption of normal anatomy or physiology [47]. The observation that the three stages of physical dependence (wanting, craving and needing) correlate with structural and functional changes in the brain establishes that physical dependence represents a disruption of normal anatomy and physiology [21]. As such, tobacco dependence can be diagnosed as a *medical condition* when symptoms of withdrawal-induced wanting, craving or needing are reported [47]. In practical terms, tobacco users fulfill the criteria for a medical diagnosis of tobacco dependence when they reach the wanting stage of physical dependence as indicated by endorsement of the first symptom on the instrument in Table 1.

While medical conditions are diagnosed on the basis of indications of altered anatomy or physiology, the psychiatric approach to diagnosis is based on indications of impairment. Under the definition offered by the DSM 5, substance use represents a mental disorder when a combination of specified symptoms causes “clinically significant impairment or distress” [7]. Dissimilar to intoxicating and illegal drugs, tobacco use rarely causes incarceration, job loss or divorce. The DSM does not explain what impairment or distress might mean in relation to tobacco use, and researchers have always assumed that anyone who satisfies the diagnostic criteria has “clinically significant impairment or distress” [48]. Table 3 lists the diagnostic criteria for Tobacco Use Disorder under DSM-5 along with some criterion-specific observations. Only two criteria must be met to satisfy the DSM-5 requirements for a psychiatric diagnosis of tobacco use disorder.

Tobacco users satisfy the DSM-5 tobacco use disorder criteria when they reach the craving stage of physical dependence. The presence of craving would satisfy criterion 4 “Craving, or a strong desire or urge to use tobacco” and smoking to alleviate that craving would satisfy criterion 11b “Tobacco (or a closely related substance, such as nicotine) is taken to relieve or avoid withdrawal symptoms.” Since all individuals at the craving stage of physical dependence smoke to relieve their craving, simply establishing that an individual has reached the craving stage of physical dependence is equivalent to a DSM-5 diagnosis of tobacco use disorder.

Although the medical and psychiatric approaches to diagnosis come from different perspectives, in practical terms, using the instrument in Table 2, tobacco dependence as a medical disorder can be diagnosed at the wanting stage of physical dependence, while DSM-5 tobacco use disorder can be diagnosed at the craving stage. The implication here is that researchers should recognize that dependence as a medical disorder may be present in individuals who do not meet DSM criteria for a psychiatric disorder; researchers should not limit their focus to individuals who meet DSM criteria.

#### Dependence onset in relation to smoking frequency

Craving after having smoked only a few cigarettes is common. [17, 28, 49, 50] In a survey of 34,000 adolescents who had tried smoking, craving or other dependence symptoms were reported by one third of those that had smoked 3 or 4 tobacco cigarettes, and by half of those that had smoked 10–19 cigarettes [28]. By the reasoning described above, half of adolescent smokers meet DSM-5 diagnostic criteria for tobacco use disorder before they have smoked a whole pack of cigarettes. This should not be surprising given the rapidity with which nicotine triggers enduring neuroplastic changes in the brains of experimental animals (see [26] for a review). Neuroplastic changes have been observed after a single dose of nicotine in animal studies [51].

Each of the first 100 cigarettes appears to promote shortening of the latencies and the appearance of additional symptoms. [17, 28, 37, 49] The prevalence of craving and other symptoms increases to about 95 % among youth who have smoked 100 or more cigarettes [17, 52, 53].

In relation to smoking frequency, symptoms of physical dependence are reported by 82 % of youth who smoke at least once per week but not every day, and by 95 % of daily smokers [52]. Consistent with reports of latencies of four weeks or more, 46 % of youth who were smoking less than once per month experienced symptoms of physical dependence [17, 29, 49, 54]. Similarly, in a national survey nearly half of youths who smoked as few as 1–3 days per month reported having experienced at least one symptom of nicotine dependence [43]. Growth in the number of symptoms of dependence reported is greatest at the lowest levels of exposure in terms of days smoking per month or cigarettes smoked per day [43]. Symptom development tends to plateau when daily consumption reaches 6–15 cigarettes [43].

#### Prognosis

The prognosis for adolescent tobacco dependence is poor. Early symptoms of dependence predict continued and escalating smoking [37]. Shortening latencies promote an escalation in smoking frequency, while more frequent smoking promotes the progression of dependence in a mutually reinforcing cycle [45, 53, 55]. The number and intensity of the symptoms experienced by very light smokers is disproportionate to their tobacco consumption, with some nondaily smokers describing their withdrawal symptoms as unbearable [56–58]. Many nondaily smokers have failed at one or more attempts to quit, and, in cessation studies, nondaily and daily smokers relapse at the same high rates [54, 59–62]. The first insights into how tobacco dependence develops came from observing that youths who used tobacco infrequently reported significant difficulty in stopping their use. It might be a fruitful first approach to ask infrequent users of other substances if they have ever failed in an attempt to stop using the drug.

Because relapse rates are very high even before the onset of daily use, craving is a grave prognostic indicator even at minimal levels of tobacco use [59, 60]. Youth become aware of craving, on average, when they are smoking two cigarettes per week [59, 60]. In a 12-year longitudinal study, smoking two cigarettes per week at age 12 increased the risk of progressing to heavy adult smoking with an odds ratio of 174 [38, 63]. The typical smoker averages more than one quit attempt per year [64], and yet current smokers are almost as numerous as former smokers in the US. As half of lifelong smokers die prematurely from their smoking [65], the appearance of craving when adolescents are smoking two cigarettes per week carries a poor prognosis for living out a normal life span.

#### Theoretical implications

The incentive-sensitization theory and others have focused attention on reward mechanisms as the primary driver of addiction [66, 67]. The concept is that psychostimulant drugs stimulate pleasure centers in the brain triggering a release of dopamine. Through repeated pairing of drug cues with the release of dopamine, the cues take on excessive salience through a mechanism of conditioned learning. Cue exposure then becomes a primary motivator for continue drug use. Nicotine does not fit the description of a psychostimulant: most smokers say it relaxes them. The sensitization-homeostasis theory provides a theoretical framework that is entirely consistent with the material presented in this article [26, 68, 69]. Under this theory, the primary site of action of nicotine is not a pleasure center, but a satiety circuit. Homeostatic mechanisms develop to oppose the action of nicotine (an opponent process model). Homeostasis is restored through neuroplastic mechanisms reacting to imbalances in neural activity. While the direct action of nicotine is to inhibit craving, the homeostatic adaptations, when unopposed by nicotine, trigger craving whenever the individual goes too long without using tobacco. Once the homeostatic adaptations have developed, the brain requires nicotine to quell craving and maintain homeostasis. Only when nicotine is required to restore brain homeostasis do smoking cues take on a special relevance. Given the differences between nicotine addiction and other forms of addiction to be described next, it is plausible that addiction to different classes of drugs may develop through somewhat different processes.

#### How tobacco differs from other drugs

As tobacco was not generally recognized as an addictive substance in the past, public health advocates have highlighted the similarities between tobacco and other addictive substances [70]. However, the differences between tobacco dependence and other forms of addiction may be important when seeking to generalize the above observations to other substances.

In the author’s opinion, the DSM 5 tolerance criteria 10a “A need for markedly increased amounts of tobacco to achieve the desired effect” (Table 3) is rarely applicable to tobacco unless the desired effect is nausea. A single cigarette remains the standard dose of nicotine from the first cigarette to the last, and novice smokers obtain the same dose of nicotine per cigarette as do adult smokers [71]. In this regard, tobacco differs from alcohol and opiates, as individuals who are addicted to these substances tend to increase their dosing over time, often to levels that would be fatal to novice users.

Tolerance to many of the effects of nicotine are unrelated to addiction [72]. The only form of nicotine tolerance that has been shown to correlate moderately with addiction is the latency to withdrawal [27, 29]. (Individuals can become tolerant to the analgesic effects of opiates without addiction being present.) As the latencies shorten from weeks to days to hours to minutes, a single cigarette becomes much less effective at sustaining the asymptomatic phase of withdrawal, which indicates “a markedly diminished effect with continued use of the same amount of tobacco” (Table 3, criterion 10b). Individuals who are addicted to alcohol or opiates develop tolerance to the intoxicating properties of the drug. Since nicotine does not cause intoxication, this form of tolerance is not a factor in tobacco dependence. As the latency to withdrawal shortens, smokers do not have to obtain more nicotine each time they smoke, but they do need to smoke at more frequent intervals.

Binging in relation to drinking refers to consuming a specified number of drinks at one time to get intoxicated. Individuals addicted to alcohol or cocaine find that one dose provokes craving for another [2]. Binging does not occur with tobacco [73]. There is no comparable phenomenon with tobacco as each cigarette decreases the urge to smoke by aborting withdrawal. When there are no restrictions on smoking, the frequency of use is remarkably constant from one day to the next and often from one decade to the next.

The DSM does not require physical dependence to make a diagnosis of drug addiction. Although it would be difficult to prove an absolute, it appears likely that tobacco dependence always involves physical dependence. In longitudinal studies, withdrawal-induced craving is a very early appearing symptom with a very high rate of endorsement [59]. Symptoms of psychological dependence and responsivity to smoking cues develop in parallel with physical dependence [74].

Although some smokers report that tobacco withdrawal causes hand tremors, the symptoms of physical dependence on tobacco are mostly psychological: impatience, irritability, anger, bad mood, restlessness, insomnia, agitation and difficulty concentrating. Unlike withdrawal from depressant drugs, tobacco withdrawal is not life-threatening. While people undergoing tobacco withdrawal may be poor company, tobacco withdrawal rarely prevents a person from fulfilling role obligations.

Many individuals who are addicted to alcohol never experience delirium tremens [3]. Those who do may experience delirium tremens on one hospital admission but not another. Patients who have experienced delirium tremens can avoid it by tapering their drinking. In contrast, all or almost all individuals with tobacco dependence are physically dependent. Once physical dependence has developed, withdrawal is unavoidable; the same symptoms appear whenever an individual goes too long without using tobacco. While it is currently believed that only the heaviest consumers of alcohol experience alcohol withdrawal, this is not true of smokers as the average frequency of use at the emergence of physical dependence is two cigarettes per week [59, 60].

Cocaine craving has been described as intense during a binge and the early withdrawal period but non-existent during the middle and late crash phases [2]. Nothing similar occurs with tobacco. During withdrawal, the desire to use tobacco grows stronger with the passage of time, waning only after several days, and sometimes persisting at low levels for a lifetime [75].

In consideration of the differences in use patterns for different drug classes it seems that opiate addiction most closely resembles that of tobacco. Patterns of sustained nondaily use termed “chipping” has been described for both classes [41, 76, 77].

## Conclusions

### Implications for other forms of drug addiction

The natural history of tobacco dependence is now fairly well established, at least in regard to its onset. As tobacco does not produce intoxication, it is clear that intoxication is not an essential quality for an addictive drug. Even without the allure of intoxication, the continuation rates for tobacco use are very high in comparison to other addictive substances: an adolescent would need to smoke only four cigarettes to have a 90 % chance of smoking for decades [78]. Whether it is measured using medical or psychiatric criteria, the prevalence of dependence among tobacco users is very high in comparison to that for drugs such as marijuana and alcohol. The high continuation rates for tobacco use likely reflect the rapidity with which symptoms of dependence emerge. Until the earliest symptoms of dependence on other drugs are identified it will not be possible to determine how quickly dependence on other drugs can develop.

No minimum in terms of amount or frequency of tobacco use has been identified that does not carry a risk of dependence for the most vulnerable individuals. Research on other substances might benefit from case studies focused on identifying the amount and frequency of use when users of those substances first felt “hooked.” The only way to discover the natural history of the development of dependence is through the experiences of those who have lived through it. In the author’s opinion, excessive skepticism regarding subjective symptoms has impeded progress. It was only through listening to the personal experiences of adolescents that the natural history of tobacco dependence was revealed.

Physical dependence on tobacco develops through three characteristic clinical stages that correlate with changes in neural density and the number of neural tracts in addiction circuitry [21, 22]. Activity in these circuits correlates with the intensity of withdrawal-induced craving. The stages of physical dependence to tobacco are recognized based only on the characteristics of the desire to smoke that is triggered by withdrawal. While different drugs produce different withdrawal symptoms, the withdrawal-induced desire to use the substance may turn out to be the common denominator. The recognition of the stages of physical dependence was made possible by distinguishing the withdrawal-induced desire to use tobacco (wanting, craving, needing) from a desire to use tobacco for other reasons (pleasure seeking, relaxation, cue-induced craving, stress-induced craving, etc.). This was done by prefacing questions with the phrase “If I go too long without smoking…” Researchers studying other drugs might find it useful to drill down on “craving” in order to distinguish withdrawal-induced desires from those arising from other motivations. It should not be assumed that desires are too subjective to be studied scientifically. Indeed, subjective reports can correlate highly with physiologic measures of brain structure and function [21, 22]. The instrument in Table 1 could be adapted to assess the withdrawal-induced desire to use other substances. The seminal work leading to the development of the instrument in Table 1 was based on interviews and focus groups with adolescent smokers who described their feelings and experiences. It might prove fruitful to repeat this exercise with individuals who use other drugs so sporadically that it is not imaginable that they could be addicted. Nobody imagined that adolescents could develop physical dependence when smoking a few cigarettes per month.

Researchers should investigate whether physical dependence to other drugs also develops through defined clinical stages. If so, these stages could be correlated with measures of neural structure to determine if other drugs change the brain in the same ways and locations as tobacco [21, 22]. Differences in the volumes of brain structures have been identified in relation to the use of other drugs, but it is not possible to relate these to the progression of dependence because progressive stages in the development of physical dependence to other drugs have not been identified, and they won’t be if researchers focus only on those individuals who have reached the final stage.

Admittedly, a “transient, mild desire” does not conform to anybody’s conceptualization of drug addiction. Yet, the earliest recognized symptom of physical tobacco dependence is a mild desire to smoke that recurs anytime the individual goes too long without using tobacco. Individuals at the “wanting” stage of physical dependence show striking differences from nonsmokers in measures of neural structure [21, 22]. It would be a mistake to assume that mild symptoms indicate trivial processes. It may be that the earliest signs of developing physical dependence for other drugs are just as mild and easily overlooked. Researchers should at least temporarily put aside preconceptions of ‘addiction’ and ‘dependence’ based on current word connotations and see where the science takes them. A predictable mild desire to use a substance when one has gone too long without might represent budding addiction. It seems entirely possible that a mild desire to use opiates one month after initial experimentation with the drug could be an indication of physical dependence. Only research will tell, but we should not ignore the possibility just because an occasional mild desire to use a drug does not match current definitions of addiction and dependence.

Smoking two cigarettes per week increases the risk of heavy adult smoking 174 fold. At least in regard to tobacco, dependence does not begin with steady daily use; it ends with steady daily use. Research on the development of dependence on other drugs might profitably focus on those considered to be casual users.

The very long latencies to withdrawal (four weeks) at the onset of physical dependence were unexpected as nicotine has a half-life of only a few hours. The latency to withdrawal in relation to other drugs should be investigated.

In summary, drug addiction research might benefit from a search for clinical stages in the development of physical dependence and a study of the latency to withdrawal. Listening receptively to the experiences of individuals who use substances infrequently may prove to be enlightening.
